# What Happens When I Watch a Ballet and I Am Dyskinetic? A fMRI Case Report in Parkinson Disease

**DOI:** 10.3389/fpsyg.2020.01999

**Published:** 2020-08-07

**Authors:** Sara Palermo, Rosalba Morese, Maurizio Zibetti, Alberto Romagnolo, Edoardo Giovanni Carlotti, Andrea Zardi, Maria Consuelo Valentini, Alessandro Pontremoli, Leonardo Lopiano

**Affiliations:** ^1^Department of Neuroscience, Center for the Study of Movement Disorders, University of Turin, Turin, Italy; ^2^European Innovation Partnership on Active and Healthy Ageing, Bruxelles, Belgium; ^3^Faculty of Communication, Culture and Society, Università della Svizzera italiana, Lugano, Switzerland; ^4^Institute of Public Health, Faculty of Biomedical Sciences, Università della Svizzera italiana, Lugano, Switzerland; ^5^Dipartimento di Studi Umanistici, University of Turin, Turin, Italy; ^6^Neuroradiology Unit, Azienda Ospedaliera Universitaria “Città della Salute e della Scienza di Torino”, Turin, Italy

**Keywords:** Parkinson’s disease, mirror neuron system, fMRI, action observation, thalamus, DLPFC, superior precentral gyrus, case report

## Abstract

**Background:**

The identical sets of neurons – the mirror neuron system (MNS) – can be activated by simply observing specific, specific movements, decoded behaviors and even facial expressions performed by other people. The same neurons activated during observation are those recruited during the same movements and actions. Hence the mirror system plays a central role in observing and executing movements. Little is known about MNS in a neurodegenerative motor disorder, such as Parkinson’s Disease (PD) is.

**Methods:**

We explored the neural correlates potentially involved in empathy and embodiment in PD through complex action observation of complex behaviors like the choreutical arts. An integrated multidisciplinary assessment (neurological, neuropsychiatric, and neuropsychological) was used for the selection of the PD candidate for the neuroimaging experimental acquisition. For the first time in literature the famous Calvo-Merino’s paradigm was administered to a PD subject.

**Key Points:**

Functional magnetic resonance imaging (fMRI) exploratory analysis shows the recruitment of the left thalamus, the right dorsolateral prefrontal cortex, and the bilateral superior precentral gyrus (one of the main hubs of the MNS). If the observed choreic movement becomes part of the observer’s motor repertoire experience, mirror neurons might activate stimulating affective empathy and making the understanding of movement an own proper body experience (cognitive embodiment).

**Main Lessons:**

Our study sheds light on a possible use of complex action observation to improve or slow the deterioration of motor abilities and levodopa-induced dyskinesias in PD patients. Indeed, the modulation of the neural area involved in complex action observation could be considered a promising target for neuro-rehabilitative intervention mediated by the elicitation of the MNS.

## Introduction

Humans are eminently social animals whose life depends on the ability to infer what others do, understanding intentions and interpreting feelings. With the discovery of the mirror neuron system (MNS), it has been shown that people do not understand others only and exclusively with cognitive associative neuronal circuits – as had been believed so far – but that a more immediate, direct, visceral understanding of the relationship between individuals exists and it is linked to particular resonance circuits – formed precisely by the MNS (Rizzolatti and Luppino, 2001; Rizzolatti and Craighero, 2004; Rizzolatti and Sinigaglia, 2006; Rizzolatti and Rozzi, 2018).

**FIGURE 1:**
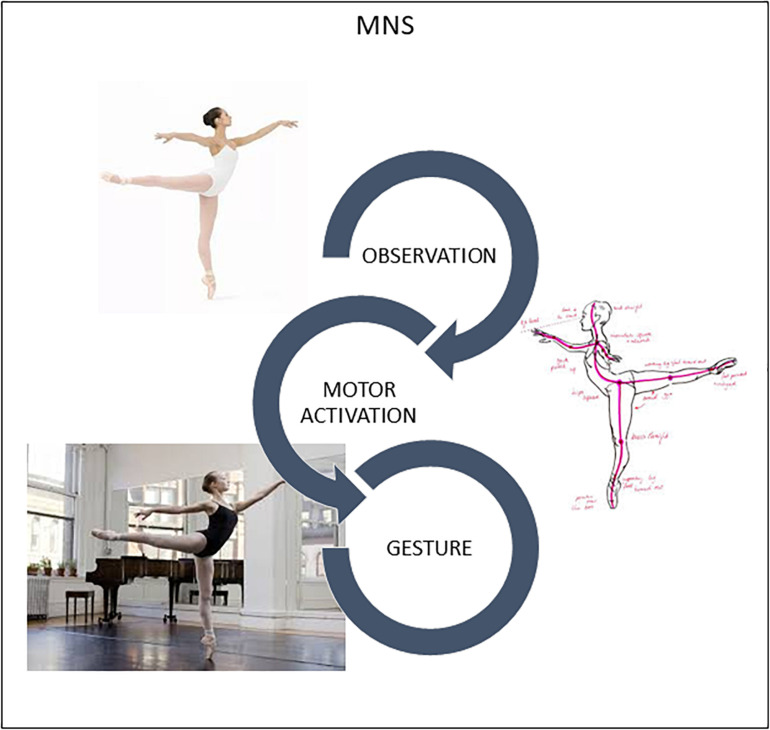
Humans are “natural born imitators.” MNS translates visual perception of another’s gesture into stimulation of this same gesture in the observer’s brain, specifically in the motor and premotor cortices. Slight activation occurs in neurons and muscles, training the brain in movement.

Indeed, recognition of other’s goal-directed motor behavior mediated by brain processes of internal simulation is the core function of the MNS: mirror neurons “understand” before being stimulated by higher cognitive circuits according to the classic scheme: perception → cognition → movement (Rizzolatti and Craighero, 2004; Rizzolatti and Sinigaglia, 2006; Rizzolatti and Rozzi, 2018). Traditionally, this algorithm was reserved to the associative areas of the neocortex that translated sensory information into motor commands (Fogassi et al., 2005; Rizzolatti and Rozzi, 2018). MNS is already recruited when a subject sees a motor action (Figure 1). The only condition is that for the observer the action is part of his motor repertoire. If the observed action is not part of the observer’s motor repertoire, mirror neurons do not activate and the understanding of movement does not become a bodily experience and, therefore, its understanding can only take place through a rational pathway (Rizzolatti and Sinigaglia, 2006).

Considering the above, the existence of the MNS provides for the existence of a phenomenon of “immediate neuronal resonance” because the observer recruits the same neuronal areas that are activated in the brain of those who perform the action (Rizzolatti and Luppino, 2001; Rizzolatti and Craighero, 2004; Rizzolatti and Sinigaglia, 2006; Rizzolatti and Rozzi, 2018). MNS properties – while being innate – rely on personal motor repertoire and can be modified by experience (Calvo-Merino et al., 2005, 2006). Furthermore, mirror neurons connecting directly to the subcortical structures of emotion can be decisive in “empathy” (Gallese et al., 2004; Singer et al., 2004; Ramachandran and Oberman, 2006; Cattaneo et al., 2007; Cattaneo and Rizzolatti, 2009). As expressed by Ramachandran (2012), the MNS «*appear to be the evolutionary key to our attainment of full culture for the way in which they allow humans to adopt each other’s point of view and empathize with one another*» (2011: xv-xvi; chapter 4). The MNS discovery has thus made possible a new conception of the motor system, also opening the way to the neurophysiological and neuroimaging investigation of issues that had previously been the exclusive preserve of the Humanities. Using these techniques, it has been shown that the mirror system consists of two large regions: the inferior parietal lobule and the ventral premotor area, to which the inferior frontal gyrus is partially associated (Rizzolatti and Luppino, 2001; Rizzolatti and Craighero, 2004; Fogassi et al., 2005; Rizzolatti and Sinigaglia, 2006; Rizzolatti and Rozzi, 2018). Today, the MNS activation can therefore be considered a sort of bioindicator of human competences.

An interesting problem was to shed light on the relationship among MNS recruitment, the personal motor repertoire and social competences, during the observation of complex behaviors like the choreutical arts. In a famous neuroimaging study by Calvo-Merino et al. (2005) the intensity of the activation of the MNS in classical ballet dancers, capoeira experts and people who had never danced was examined. The aim of the research was to establish whether the brain areas pertaining to the MNS were activated differently according to the subjects’ dance experience. The authors suggested that the MNS «*integrates observed actions of others with an individual’s personal motor repertoire and suggest that the human brain understands actions by motor simulation*» (Calvo-Merino et al., 2005; page 1243). In a second research, Calvo-Merino et al. (2006) found «*greater premotor, parietal, and cerebellar activity when dancers viewed moves from their own motor repertoire, compared to opposite-gender moves that they frequently saw but did not perform*» (page 1905). The authors concluded that humans understand motorically actions not only from a visual point of view (Calvo-Merino et al., 2006). Indeed, the most important function of the mirror system seems to be detection of motor patterns and recognition of the intentions of others’ actions (Rizzolatti and Craighero, 2004; Calvo-Merino et al., 2005).

The MNS is sensitive to neurodegeneration (Farina et al., 2020). A progressive alteration in the posterior-anterior direction and associated with initial compensatory mechanisms has been suggested in the Alzheimer’s disease continuum. Considering frontotemporal dementia and amyotrophic lateral sclerosis, MNS abnormalities seem to be able to explain language and inter-subjectivity deficits. MNS could be altered also in PD. However, motor and cognitive performances seem to be supported by MNS hyperactivation in the early stages of the disease (Farina et al., 2020). To analyze neurodegenerative diseases considering MNS findings allows to better understand the clinical manifestations and attempt new rehabilitation approaches (Palermo et al., 2019a; Farina et al., 2020).

Alternative therapeutic interventions – such as dance therapy – are based on the link between MNS, empathy, motor, and social skills. Dance therapy opens to emotional listening through the movement of the body, stimulating a creative process that favors the improvement of relational dynamics, existential enrichment, and patient resilience. Dance therapy is a non-invasive, simple treatment option, which promotes gait, motor function, cognition, and mental symptoms in Parkinson’s disease (PD) (Hashimoto et al., 2015; Pereira et al., 2019). Importantly, it is beneficial in improving executive functions (Zhang et al., 2019), the damage of which has previously been associated with dyskinesias-reduced-self-awareness (Amanzio et al., 2014; Palermo et al., 2017a, 2018a,b; Palermo and Morese, 2018c) and impulse control disorder (Palermo et al., 2017b). In such cases, even if patients do not complain about involuntary movements, they can have a deleterious effect on their own motor repertoire.

There is increasing evidence supporting the use of complementary therapies based on action observation as means to potentially benefit PD (Palermo et al., 2019a). Nonetheless, little is known about the mirror system in PD. Some evidence for altered brain activation in mirror neuron areas has been previously found (Alegre et al., 2011; Pohl et al., 2017), but considering only Theory of Mind. The first endpoint of our single case was to start exploring our hypothesis on involvement of MNS components in PD. We aimed to have supporting information about (1) the neural correlates associated with patients’ visual participation in the choreic arts; (2) the possible value of action observation in clinical practice. Our secondary endpoint was to evaluate the feasibility of the functional neuroimaging research protocol on patients with movement disorders – modifying where necessary acquisition parameters and procedures – prior to starting experimenting on a larger sample.

Specifically, we reproduce here the paradigm presented by Calvo-Merino et al. (2005) since it is suitable for verifying possible alteration of the “proprioceptive-control mirroring effect” in chronic motor impairment. Importantly, this approach was attempted for the first time in Literature on a patient with movement disorders.

## Materials and Methods

We started recruiting patients at the Center for the Study of Movement Disorders, Department of Neuroscience, University of Turin, Italy. The selection procedure was conducted among the patients taken in charge by the Unit for possible access to advanced therapy, in terms of deep brain stimulation intervention (Amanzio et al., 2014; Palermo et al., 2017a, 2018a,b). Twenty patients were considered as possible candidates.

### Inclusion and Exclusion Criteria

Inclusion criteria were: (a) A good clinical response to levodopa with the presence of peak-of-dose dyskinesias and wearing off or on-off phenomena; (b) Stable treatment regimen for at least 6 months and aimed at treating each patient optimally; (c) at least elementary school education (d) Mini Mental State Examination (Folstein et al., 1975) uncorrected score ≥27 as per medico-legal evaluation guidelines (De Vresse et al., 2014) and in order to include only a cognitively non-impaired subject (Amanzio et al., 2014; Palermo et al., 2017a, 2018b); (d) Willing to participate in the study and acquisition of a written informed consent.

Exclusion criteria were: (a) random on-off; (b) early morning and painful dystonia; (c) behavioral abnormalities such as major depression, dysthymia or alexithymia based on DSM-V criteria (American Psychiatric Association [APA], 2013); (d) past and present neurological disorder and/or brain organic conditions (other than PD); (e) pharmacological therapies that could directly impact cognitive functioning, other than dopaminergic pharmacological replacement treatment; (f) any contraindications for participation in a neuroimaging exam (e.g., claustrophobia, implants, metal splinters,….).

A first voluntary subject was recruited based on these criteria to carry out a first piloting on the feasibility of the fMRI study on PD patients.

### Physical Setting

Patients were admitted in week-in-hospital at the Parkinson’s and Movement Disorders Unit (*Città della Salute e della Scienza di Torino* Hospital) belonging to the Department of Neuroscience. The neurological examination was carried out in the inpatient ward, in the patient’s bed and in rooms properly equipped and able to guarantee privacy.

The fMRI experimental session was conducted on a 3T Philips Ingenia scanner (Neuroscience Institute of Turin – Neuroimaging Centre) located at the same hospital. The pre-scan interview and instructions were given in the preparation room; the training was carried out in the scanner room allowing the subject to become familiar with the instrumentation and the experimental setting before starting the experimental acquisition.

### Procedures

All the procedures were carried out on a week-in-hospital basis, being most of them part of the normal evaluation for the selection of candidates for brain surgery.

Neurological evaluation was performed both in the absence of drug therapy and over the course of the maximum-benefit-peak of the first daily dose (Amanzio et al., 2014; Palermo et al., 2017a, b). Neuropsychological evaluation was performed on the second day of hospitalization in the on-state and it lasted about an hour and a half. On the third day of hospitalization, the functional magnetic resonance imaging (fMRI) study was performed. Importantly, the patient was in therapeutic washout during neuroimaging acquisition, to avoid possible confounding effects of dopamine treatment effects on neuroimaging results (Palermo et al., 2017a, 2018b). Indeed, the last pharmacological administration was performed 5 h before the fMRI experimental session.

Procedural fidelity was evaluated in each phase. For this purpose, the entire protocol was divided into successive steps, of which the following occurred: presence of dedicated operators, compliance with the daily scheduling, execution, qualitative evaluation of the acquired parameters, patient’s collaboration.

### Neurological, Neuropsychiatric and Neuropsychological Assessment

With specific reference to the patient selection for the piloting study in fMRI, neurological evaluation was transmitted by a neurologist blind to the aim of the study who had previously used the Movement Disorder Society – Unified Parkinson’s Disease Rating Scale (MDS-UPDRS) to provide a clinimetric evaluation of patients’ clinical profile (Antonini et al., 2013). Specifically, motor features and disease severity were assessed using MDS-UPDRS part III and UPDRS total scores, while dyskinesias were assessed using MDS-UPDRS part IV (for both On-/Off- conditions). Hoen and Yahr’s scale (H&Y) was used to outline the disease stage (Hoehn and Yahr, 1967).

The neuropsychological assessment was performed in line with previous researches (Amanzio et al., 2014; Palermo et al., 2017a, 2018a, 2018b) and based on the guidelines of the Task Force commissioned by the Movement Disorder Society to identify Mild Cognitive impairment (Litvan et al., 2012). These criteria represent an operating scheme that evaluates the cognitive profile on two levels differing in their methods of evaluation and diagnostic certainty (Litvan et al., 2012). For this case report we applied the first level of evaluation.

Neuropsychiatric assessment included the Hedonistic Homeostatic-Dysregulation scale (HHD), the Beck Anxiety Inventory (BAI), the Beck Depression Inventory (BDI), the Apathy Scale (AS), the Young Mania Rating Scale (YMRS), and the Brief Psychiatric Rating Scale 4.0 (BPRS 4.0) (Amanzio et al., 2014; Palermo et al., 2017a, 2018a, 2018b).

The neuropsychological battery included the Mini-mental State examination (MMSE) and the Addenbrooke’s Cognitive Examination – Revised version (ACE-R) to detect the presence of a general cognitive deterioration; attention, perceptual tracking of a sequence and speeded performance were analyzed using the Attentional Matrices (AM) and the Trail Making Test part A (TMT-A); abstract reasoning and fluid intelligence using the Colored Progressive Matrices (CPM-36); executive functions using the Frontal Assessment Battery (FAB), Trail Making Test part B and part B-A, and the Wisconsin Card Sorting test (WCST); short-term and working memory abilities using Rey-15 word test and Digit Span (backward and forward, respectively). Lastly, information retrieval was evaluated using the Phonemic Fluency Test – letters F, A, S (FAS) (Palermo et al., 2017a, 2018a,b).

### fMRI Data Acquisition and Analyses

Anatomical images were recorded using a T1-weighted sequence (TI = 1650 ms, TR = 4.8 ms, voxel-size = 1 mm × 1 mm × 1 mm, TE = 331 ms). Functional data were collected using T2^∗^-weighted EPI (TE = 35 ms, TR = 2.20 s slice gap = 0.28 mm, FOV = 24 cm, flip angle = 90°, slices aligned on the AC-PC line, slice-matrix = 64 × 64). The patient watched 24 classical ballet videos performed with difficult movements [dance movements (3 s) followed by jittery fixation cross (5–7 s)] and 24 videos of daily movements [walking (3 s) followed by jittery fixation cross (5–7 s)]. To avoid lack of attention, each participant had to randomly estimate (4 s) the difficulty of the last figure seen on a Likert scale (range 0–4). Image preprocessing was performed using SPM12 (Wellcome Department of Cognitive Neurology, London, United Kingdom). Realignment of functional images were spatially applied to the first volume, coregistration of anatomical images were processed to the mean of them. Normalization of the functional images to the MNI space and smoothing (8 mm) were performed. We applied a General Linear Model to convolve subjects’ responses with canonical hemodynamic response.

## Case Report

A 59-years-old woman with diagnosis of idiopathic PD was admitted to the hospital for the ascertainment of requirements for subthalamic nucleus (STN)- deep brain stimulation surgery.

The patient had 5 years education. She had normal developmental milestones and no medical history of note. She had a 12-year PD story, with negative family history. Onset of neurological disorders with tremor in the right hand followed by gradual motor hindrance of the upper limb. For about 4–5 years she has been reporting motor fluctuations, with frequent off-phase and dyskinesias. Over the years, levodopa therapy has been set with a gradual increase in the dosage up to 1050 mg/day.

At the time of this case report, she was retired from work. Married, with children and grandchildren, she maintains autonomy in daily living. At the clinical interview, the patient is alert and collaborative, oriented in time and space. She had no alterations in mood and motivation.

In addition to the normal evaluation procedures, it was verified that she met the requirements for access to the experimental study. She agreed to undergo the functional magnetic resonance imaging paradigm in addition to all the exams scheduled during hospitalization.

Motor features and disease severity were evaluated in on-/off- conditions (MDS-UPDRS on = 61; MDS-UPDRS off = 99; MDS-UPDRS part III on = 10; MDS-UPDRS part III off = 49; MDS-UPDRS part IV on = 4; MDS-UPDRS part IV off = 4; H&Y on = 0; H&Y off = 2). The neuropsychological assessment was performed in the best-on phase, immediately after the neurological examination and the approval by the treating neurologist.

The patient exhibited a normal global cognitive profile, reaching normative scores on all the neuropsychological batteries, however, slight abnormalities were detected for the performance on short-term memory for unstructured verbal material (Table 1). The neuropsychiatric assessment revealed no behavioral changes.

**TABLE 1:** Neuropsychiatric and neuropsychological assessment in the on-phase of the disease.

Assessment		Scoring	Cut -off
Age (years)		59	
Education (years)		5	
*Neuropsychiatric assessment*			
AS	[42]	6	≤14
BDI	[39]	4	≤10
BAI	[63]	4	≤21
YMRS	[44]	2	≤12
BPRS 4.0	[168]	26	
HHD	[5]	1	
*Neuropsychological assessment*			
MMSE	[30]	26.74	≥24
ACE-R	[100]	84	≥82
FAB	[18]	14.80	≥13.48
AM	[60]	29	≥31
TMT A	[500]	54	≤94
TMT B	[500]	98	≤283
TMT B-A		89	≤187
FAS		23	≥17.35
Digit Span Forward	[9]	3.75	≥3.75
Rey-15 instant word test	[75]	24.4	≥28.53
Rey-15 delayed word test	[15]	5.2	≥4.69
CPM-36	[36]	22.50	≥18.96
WCST%		53.12	≥37.1
WCST% errors		46.87	
WCST% perseverative errors		26.66	≤42.7

*Where it is possible, maximum scores for each test are shown in square brackets. Wherever there is a normative value, the cut-off scores are given in the statistical normal direction; the values refer to the normative data for healthy controls matched according to age and education. ACE-R, Addenbrooke’s Cognitive Examination - Revised version; AM, Attentional Matrices; AS, Apathy Scale; BAI, Beck Anxiety Inventory; BDI, Beck Depression Inventory; BPRS 4.0, Brief Psychiatric Rating Scale 4.0; CPM-36, Colored progressive Matrices-36; FAB, Frontal Assessment battery; FAS, Phonemic Fluency Test; HHD, Hedonistic Homeostatic-Dysregulation scale; MMSE, Mini-mental State examination; TMT, Trail Making Test; YMRS, Young Mania Rating Scale; WCST, Wisconsin Card Sorting test.*

**FIGURE 2:**
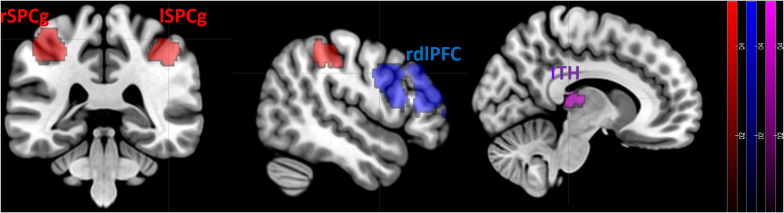
FMRI results. Differences in the neural activation between dance movements vs. daily movements. Statistical maps are displayed on a standard T1 template. lTH, left thalamus; rSPCg, right superior precentral gyrus; lSPCg, left superior precentral gyrus; rdlPFC, right dorsolateral prefrontal cortex.

During the observation of dance movements contrasted to daily movements, bold signal activation was higher in the left thalamus (lTH: *x* = −3 *y* = −22 *z* = 9, *p* = 0.002 FWE-corrected), right superior precentral gyrus (rSPCg: *x* = 40 *y* = −35 *z* = 47, *p* = 0.002 FWE-corrected), left superior precentral gyrus (lSPCg: *x* = −37 *y* = −38 *z* = 51, *p* = 0.000 FWE-corrected), right dorsolateral prefrontal cortex (rdlPFC: *x* = 47 *y* = 32 *z* = 30, *p* < 0.001 FWE-corrected) (Figure 2).

In the post-acquisition interview, the subject reported that the experience in fMRI, however, complex at the beginning, was gradually more acceptable. Seeing videos helped her not to think about the discomfort felt in the scanner. The proposed activity was not excessively difficult and left her with a pleasant feeling of well-being. She would have liked to move with such gracefulness and harmony. She felt like dancing and asked if there are dance classes for people like her.

## Discussion

*Emotion* is – as the word itself says – *movement*. Not only a metaphorical *motus* of the soul. Emotion is a reaction that underlies neurochemical changes and bodily manifestations with biological and communicative purposes. The choreutical arts fit exactly into the experience of the mind-body union (embodiment), accompanying people to fully experience their own emotional contents, express and transform them through dance. Dance therapy applied to PD has the peculiarity of proposing both a motor and emotional stimulation, in a pathology whose most significant symptoms are precisely motor impairment accompanied by behavioral abnormalities.

To experience the emotion linked to the gesture, it is not necessary to carry out the movement. The identical sets of brain areas can be activated in an individual who is simply witnessing another person performing a movement as if he/she was actually involved in the motor behavior as the one actually engaged in it (Calvo-Merino et al., 2005). The basal ganglia – whose dysfunction results in a wide range of neurological conditions including PD – may be involved in action observation, since the STN shows similar activity changes in both movement execution and observation (Marceglia et al., 2009; Alegre et al., 2010). Importantly, given the association between MNS and empathy, Theory of Mind impairment seems to be at least partially mediated by MNS dysfunction in PD (Alegre et al., 2011). This case report has been conducted to consider the appropriateness of the Calvo-Merino’s et al. (2005) fMRI paradigm applied to PD and provide information about the MNS in motor disorders. We aimed at answering the questions: What happens when a PD patient goes from observing a mere facial expression to evaluating the observation of complex behaviors like the choreutical arts? Does a phenomenon of “immediate neuronal resonance” persist for complex movements in PD? Could this mechanism be exploited for the rehabilitation of motor disorders?

We managed to make a complete acquisition of the chosen fMRI paradigm, supporting our hypothesis that it is possible to propose this experimentation to a sample of PD patients. Data collected in this circumstance suggest that brain areas connected with the MNS are activated, but the dorsolateral prefrontal cortex and the thalamus are also elicited.

Motor-related cortex, such as the precentral gyrus, has been previously identified as key neural underpinning involved in the “mirroring” of emotional expressions (Pfeifer et al., 2008). Indeed, the activity in the precentral gyrus has been proposed as regions that facilitate internal simulation as a mechanism for affective empathy (Hooker et al., 2008). Hooker et al. (2008) found that the superior portion of the precentral gyrus showed higher recruitment during tasks of social change perception that can only be overcome with an understanding of body gestures and facial expressions. Moreover, the SCP was found to be related to action observation and acquired motor skills in a study by Calvo-Merino et al. (2005): it therefore seems possible that this neural area may be involved in the generation of internal motor representation of observed action (and emotions).

The thalamus – which provides input to the premotor cortex – has been previously found to be elicited by action observation in a fMRI study aimed at examining longitudinal changes in neuronal activity in a group of patients with subacute stroke (Brunner et al., 2014). A tendency of increase in activation over time was observed in the left thalamus (Brunner et al., 2014). The thalamus has not typically been ascribed mirror properties, but it is an important relay station. Moreover, a major input to premotor areas is associated with voluntary movements (Herrero et al., 2002). Brunner et al. (2014) suggested that the strong thalamic response may be interpreted as a potential compensatory mechanism, emphasizing the importance of somatosensory feedback for functional recovery. Considering the above, the thalamus recruitment in our PD patient could therefore be attributed to the prevalence of emotional, motivational and reward aspects activated by complex action observation. Indeed, the thalamus has been previously found to be related with both mentalizing and emotion (Adolphs, 2003, 2006; Hooker et al., 2008), especially when predicting their own or others’ future emotional response (Hooker et al., 2008). The thalamus can have a role also when successful performance requires the update of action-outcome associations (Wolff and Vann, 2019).

The dlPFC has connections with the thalamus and parts of the basal ganglia (Baddeley, 1986). This brain area has direct influence on social behavior (Baddeley, 1986), since it performs cognitive control in complex social situations (Weissman et al., 2008). Specifically, dlPFC is involved in maintaining the internal representation of intentions and the norms for achieving them (Miller and Cohen, 2001), in generating and maintaining causal links between actions and their outcomes so that previous experiences can guide the selection of future behaviors (Tsujimoto and Sawaguchi, 2004, 2005; Genovesio et al., 2006). It is therefore not surprising that dlPFC is recruited by complex action observation.

We claim that complex action observation may constitute a possible access to the motor system in PD. Not only basal ganglia might be engaged by MNS activity (Alegre et al., 2010), but action observation involves both cortical and subcortical processes (Daneault et al., 2013; Caligiore et al., 2017). Before us, Brunner et al. (2014) suggested action observation as a possible gateway to retraining motor function during rehabilitation. The modulation of the neural area involved in this process could be considered a promising target for neuro-rehabilitative intervention mediated by the elicitation of the MNS. The implementation of training programs based on the observation of executed actions could allow the activation of motor representations and the reinforcement of old and/or new motor patterns learning, while modulating motivational processes in PD. Di Iorio et al. (2018) have recently suggested that action observation is a “safe and feasible” rehabilitative exercise for improving balance, gait, and reducing falls in PD. Their positive findings, the simplicity of treatment, the lack of side effects, support our hypothesis of exploiting MNS, action observation and “somato-aesthetic empathy” to act on a motivational and behavioral level. To date, yet almost nothing has been attempted against dyskinesias, which instead require prolonged monitoring and complex medical management.

Dyskinesia can have harmful effects on the quality of life of both patients and caregivers and create extra pressure on the health system (Daneault et al., 2013; Palermo et al., 2019b). While different approaches are adopted by movement disorders specialists to delay or manage levodopa-induced dyskinesias, general practitioners and neurologists without specific skills may have difficulty controlling involuntary movements, while maintaining a significant clinical improvement in the typical PD symptomatology (Daneault et al., 2013). Innovative interventions are needed to meet the unmet needs of PD patients and therapies for PD go far beyond pharmacological treatment, deep brain stimulation or stem cells (Palermo et al., 2019a). The most recent therapeutic approaches have highlighted the importance of the multidisciplinary perspective and the usefulness of physiotherapy and the so-called “complementary therapies” (Palermo et al., 2019a), among which complex action observation could give interesting results (Caligiore et al., 2017).

As rightly pointed out by Daneault et al. (2013), clinicians must consider the patient’s own perspective on the impact of levodopa-induced dyskinesias on his/her motor repertoire. Where the impact is considered not relevant, dyskinesias are not considered problematic. However, these considerations must be interpreted cautiously, considering literature showing that dyskinetic patients may suffer from Dyskinesias-Reduced-Self-Awareness (Palermo et al., 2017a, b, 2018b, 2019b; Morese and Palermo, 2020). Consequently, even if patients do not complain about their levodopa-induced dyskinesias, dyskinesia can still have a deleterious effect on their own motor repertoire. As such, slight dyskinesia may not be problematic, but more severe forms may reduce quality of life by affecting the patient’s motor repertoire (Daneault et al., 2013). Since clinicians should broaden the motor repertoire available to patients when assessing the efficacy of their treatment strategy against levodopa-induced dyskinesias, strengthening this repertoire is essential. The principle behind the reasoning is that if the observed action becomes part of the observer’s motor repertoire thanks to affective empathy, mirror neurons activate and the understanding of movement becomes a bodily experience (Rizzolatti and Sinigaglia, 2006). Indeed, complex action observation – like that involved in the observation of choreutical movements – can elicit modulatory brain processes at any level, going from peripheral districts of the body to the motor brain areas and higher-level circuits or, conversely, going from central movement preparatory areas to motor areas and the periphery of the body (Mulder, 2007; Caligiore et al., 2017).

Consequently, complex action observation might improve or slow the deterioration of motor abilities in PD patients since it is able to evoke a huger neural activation of the cortical-subcortical network that supervises motor control, partially compensating the damages of motor execution areas (Caligiore et al., 2017).

The most critical limitation to our inferences is that we only have data from a single case now. Therefore, the ability to draw generalizable assumptions is severely hindered and our conclusions must be accepted with caution. However, this study is exploratory in nature and aimed at providing first indications on the topic. Complex action observation effects over dyskinesia, motor impairment and behavioral abnormalities need to be further investigated. Future research should include a greater sample size and thoroughly evaluate MNS embodiment neural mechanisms over time using long-term follow-up.

## Data Availability Statement

The datasets generated for this study are available on request to the corresponding authors.

## Ethics Statement

The studies involving human participants were reviewed and approved by the Ethics Committee “A.O.U. City of Health and Science of Turin – A.O. Mauritian Order – A.S.L. City of Turin” as part of the basic research criteria followed by the Neurological Units. All the procedures described in the study were performed in compliance with security, integrity, and privacy. The patients/participants provided their written informed consent to participate in this study. Written informed consent was obtained from the patient for the publication of any potentially identifiable images or data included in this article.

## Author Contributions

The research was developed by SP who wrote the manuscript and dealt with the critical revision processes as PI. SP also performed the neuropsychological evaluation (organization and execution). RM operatively organized and developed the fMRI study, created the experimental paradigm, analyzed the fMRI data (execution – operative), participated in the interpretation of the results, and the writing of the manuscript. MV organized and conducted the acquisition of magnetic resonance imaging and discussed fMRI results and participated in writing of the document. MZ and AR performed the neurological evaluation (execution) and took part in the organization of the research and in the diagnostic phase (organization and clinical diagnostic evaluation). EC, AZ, and AP have provided the theoretical bases on the core arts and have created the video clips (execution, recording, and editing) used in fMRI. LL supervised the neurological evaluation and participated in the writing of the manuscript (review and criticism). All authors approved the submission of the manuscript.

## Conflict of Interest

The authors declare that the research was conducted in the absence of any commercial or financial relationships that could be construed as a potential conflict of interest.
